# The Influence of Soil Acidity on the Physiological Responses of Two Bread Wheat Cultivars

**DOI:** 10.3390/plants9111472

**Published:** 2020-10-31

**Authors:** Brigitta Tóth, Csaba Juhász, Maryke Labuschagne, Makoena Joyce Moloi

**Affiliations:** 1 Institute of Food Science, University of Debrecen, 138 Böszörményi St., 4032 Debrecen, Hungary; 2 Arid Land Research Centre, University of Debrecen, 138 Böszörményi St., 4032 Debrecen, Hungary; juhasz@agr.unideb.hu; 3 Department of Plant Sciences, University of the Free State-Main Campus, P.O. Box 339, Bloemfontein 9300, South Africa; LabuscM@ufs.ac.za (M.L.); MoloiMJ@ufs.ac.za (M.J.M.)

**Keywords:** antioxidative enzyme activity, low pH, proline, protein, wheat

## Abstract

The recent study was conducted to examine the influence of acidic soil on the activities of ascorbate (APX) and guaiacol peroxidase (POD), proline, protein as well as malon-dialdehyde (MDA) content, in two commercial spring wheat cultivars (PAN3497 and SST806) at different growth stages (tillering and grain filling). A cultivar effect was significant only for MDA content, while the treatment effect was highly significant for proline, protein, and MDA. The sampling time effect was significant for most characteristics. MDA, antioxidative capacity, as well as protein content increased with maturity. At grain filling, MDA and proline contents were significantly higher at pH 5 than pH 6 and 7 for both cultivars, with the highest content in SST806. Similarly, SST806 had significantly higher APX and POD when growing at pH 5. There were no significant differences in protein content at grain filling between either genotype or treatments affected by low pH. This study showed that growth stage and soil pH influence the rate of lipid peroxidation as well as the antioxidative capacity of wheat, with a larger effect at grain filling, at pH 5. Although SST806 had higher proline, POD, and APX content than PAN3497 at this growth stage, this coincided with a very high MDA content. This shows that the high antioxidative capacity observed here, was not associated with a reduction of lipid peroxidation under low soil pH. Further research should, therefore, be done to establish the role of the induced antioxidant system in association with growth and yield in wheat.

## 1. Introduction

Climate change has severe consequences on the natural environment and agricultural production, leading to poverty and food insecurity [1]. The indirect impact of such alterations include a shift in cropland distribution, while a direct effect involves, among others, sulfidic wetland drying (because of climate change-induced drought). As a result, climate change has a catalytic effect on the changes in soil quality [2], specifically soil acidification, which is a worldwide problem, but especially so in Africa and Europe. Africa contributes a higher proportion (16.7%) of global acidic soils compared to Europe (9.9%) [3,4]. In parts of South Africa, for example, the KwaZulu-Natal province, 85% of soils have a pH of less than five [5], which has a significant impact on crop production, leading to yield losses of up to 70% [6]. The extent of yield reduction depends on the level of hydrogen ions (which affects plant growth and development) [7,8,9], climatic conditions and genetic background of the cultivar. In addition, the combination of H^+^-toxicity, nutrient deficiency and reduced water uptake influences growth negatively [10]. Many studies associate soil acidification with nitrogen fertilizer over-application [11,12,13], acid rain [13,14,15], changes in soil physical properties [16], plant residues [17], and changes in soil chemistry [18] as the main common reasons worldwide. 

Acid soil affects phosphorus uptake, root length, and mean root diameter [19]. Furthermore, low soil pH could influence the levels of reactive oxygen species (ROS) [20] such as singlet oxygen (^1^O_2_), superoxide anion (O_2−_), hydrogen peroxide (H_2_O_2_), and hydroxyl radical (OH). These radicals are able to oxidize vital cellular ultrastructure and give rise to oxidative damage and devastation of cellular organelles [21]. For instance, Zhang et al. [22] reported that lipid peroxidation and H_2_O_2_ concentration increased in rice seedling when H^+^ concentration was high. Similarly, growing *Plantago* in pH 4 soil contributed to enhanced lipid peroxidation [23]. Yang et al. [24] indicated that low soil pH enhanced the membrane permeability in Eucalyptus leaves. Contrary to this, pH 4 did not affect H_2_O_2_, malondialdehyde (MDA), electrolyte leakage, and protein oxidation in the roots and leaves of *Plantago algarbiensis* and *P. almogravensis* [25].

To compensate for the unfavorable influence of free radicals, plants possess an antioxidative system (non-enzymatic and enzymatic) aimed at reducing the effect of oxidative stress [26,27]. The activity of antioxidant enzymes was associated with low soil pH in cucumber, citrus, and rice [22,28,29]. Soil acidity selectively increased ascorbate peroxidase (APX) while it reduced superoxide dismutase (SOD) and catalase (CAT) activity in rice roots [22]. Such selective induction of antioxidative enzymes (monodehydroascorbate reductase, guaiacol peroxidase, APX, and glutathione reductase) was also observed in cucumber [28]. In some instances, however, increase in the antioxidative enzyme activities was not sufficient to protect plants against oxidative damage [23]. Results of a study in the leaves and roots of *P. algarbiensis* and *P. almogravensis* contradicted such findings, where the antioxidative enzymes were not affected by low soil pH [25,30].

To date, most studies have focused on the influence of soil acidity on plant growth and yield. There is limited knowledge of the influences of low soil pH on the physiological aspects of wheat, especially on leaves. Therefore, our experiment was set to investigate the influence of low soil pH on lipid peroxidation, protein and proline contents as well as on antioxidant enzymes (ascorbate oxidase and guaiacol peroxidase) activities, in the leaves of two South African hard spring wheat cultivars, at two growth stages, tillering and grain filling.

## 2. Results

**Figure 1:**
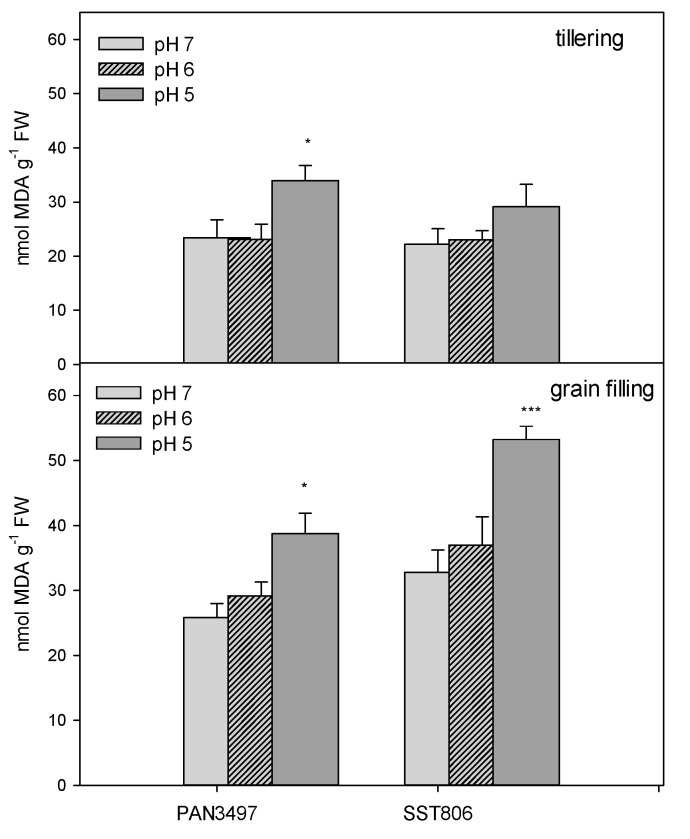
The MDA content of PAN3497 and SST806 grown at three soil pH levels (pH 7, 6, and 5), with two sampling times (tillering and grain filling). Values are the averages of three biological and technical repetitions ± SE. Significant difference compared to pH 7: * *p* < 0.05, *** *p* < 0.001.

The rate of lipid peroxidation was measured by the amount of MDA used in plants grown in low pH soil. Compared to the pH 7 treatment, the highest differences in MDA content were observed at pH 5 for PAN3497 (34%) and SST806 (39%) at the grain filling stage, while at tillering, significantly higher levels of MDA was recorded in PAN3497 only. The levels of lipid peroxidation were higher at grain filling than the tillering stage for both genotypes. For both growth stages and genotypes, the pH 6 treatment was not significantly different from pH 7 (Figure 1). 

**Figure 2:**
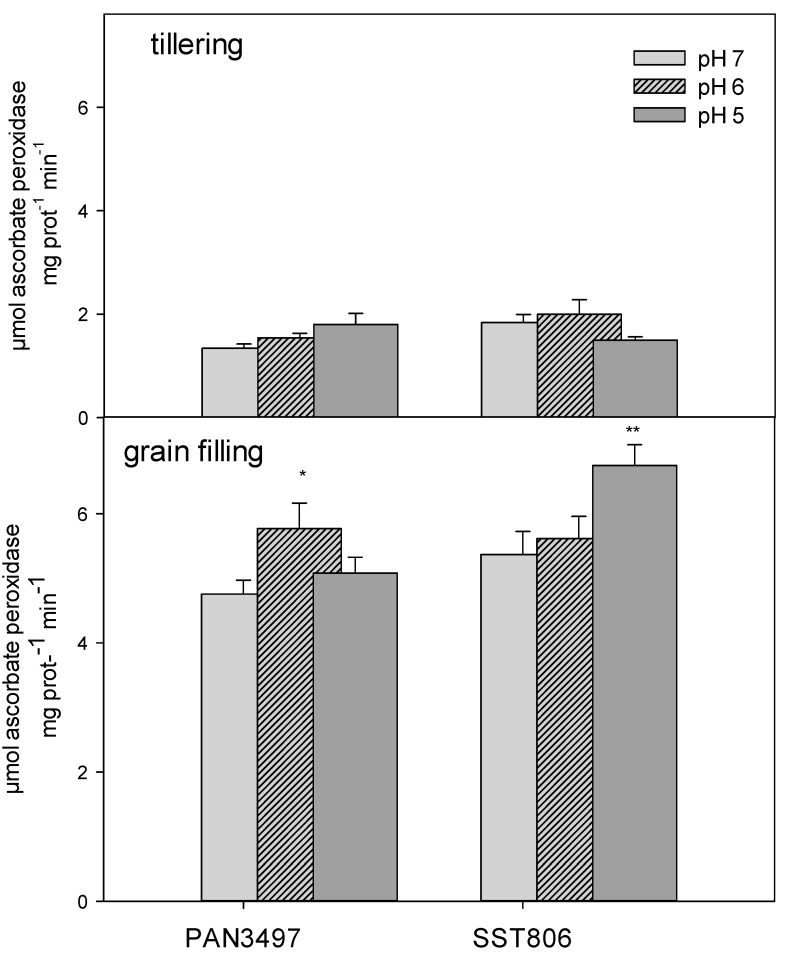
The APX activity of PAN3497 and SST806 grown at three soil pH levels (pH 7, 6, and 5), with two sampling times (tillering and grain filling). Values are the averages of three biological and technical repetitions ± SE. Significant difference compared to pH 7: * *p* < 0.05, ** *p* < 0.01.

At grain filling, cultivars responded differently to pH treatments, where PAN3497 had a significant increase in APX activity at pH 6 while SST806 had highly significantly increased activity at pH 5. In contrast to this, at the tillering stage, APX activity did not change significantly for any of the pH treatments and genotypes (Figure 2). 

**Figure 3:**
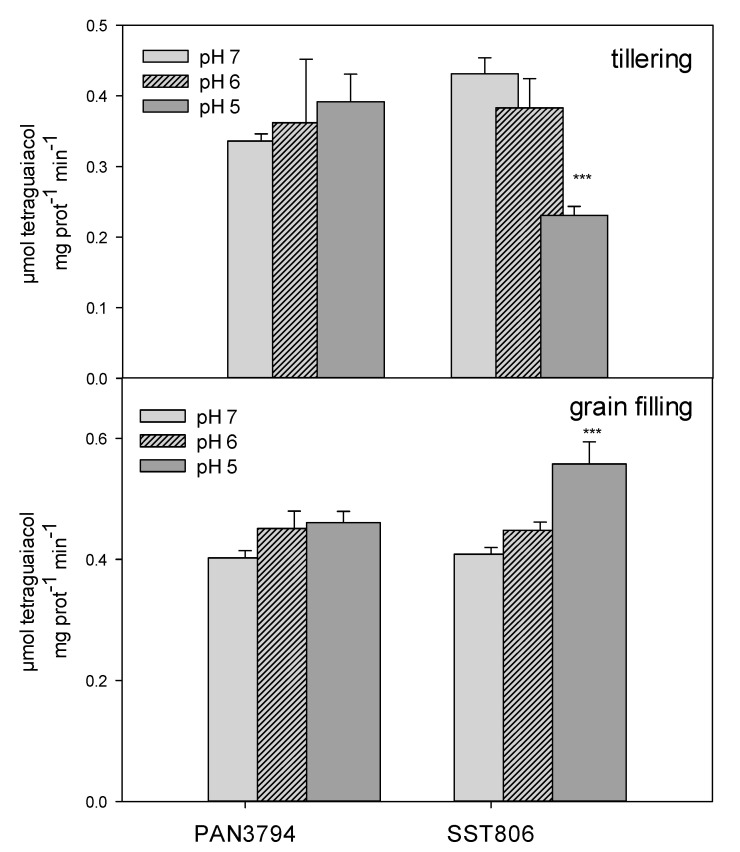
The peroxidase (POD) activity of PAN3497 and SST806 grown at three soil pH levels (pH 7, 6, and 5), with two sampling times (tillering and grain filling). Values are the averages of three biological and technical repetitions ± SE. Significant difference compared to pH 7: *** *p* < 0.001.

Higher tetraguaiacol content (representing high peroxidase activity) was measured during grain filling than tillering (Figure 3). Peroxidase activity for PAN3794 showed no significant changes for either growth stage. Contrary to this, a highly significant reduction in peroxidase activity was observed for SST806 at pH 5 during tillering. However, at grain filling, activity was significantly higher at pH 5.

**Figure 4:**
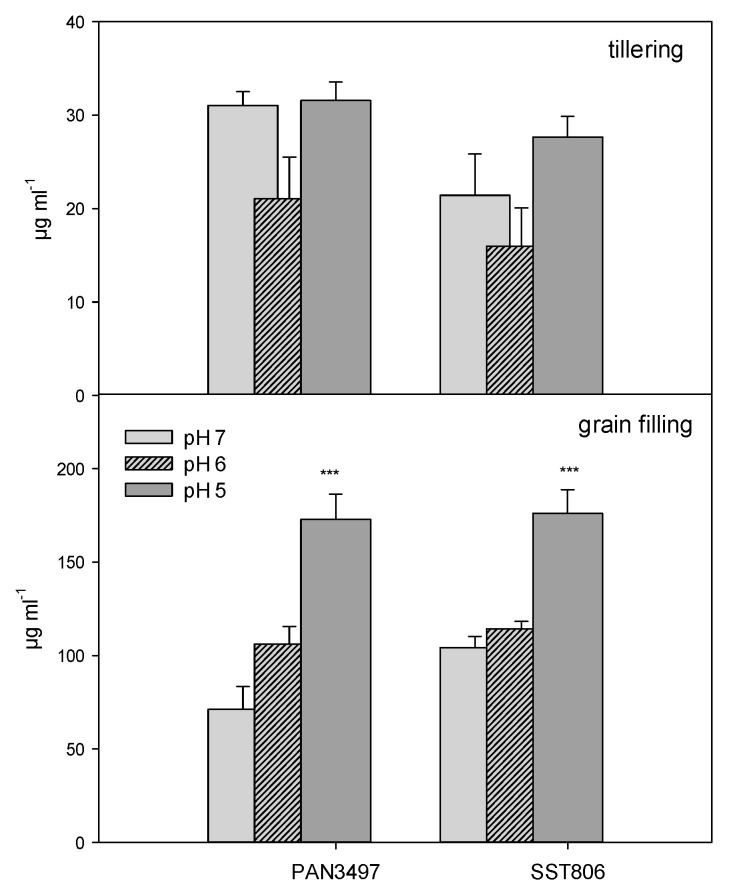
Proline content in leaves of PAN3497 and SST806 grown at three soil pH levels (pH 7, 6, and 5), with two sampling times (tillering and grain filling). Values are the averages of three biological and technical repetitions ± SE. Significant difference compared to pH 7: *** *p* < 0.001.

Proline content increased with a decrease in the pH for both genotypes at grain filling. This increase, however, was significant only at pH 5. The pattern, however, changed at the tillering stage where pH 6 influenced proline accumulation negatively. For PAN3497, there were no significant differences between pH 7 and 5. SST806 displayed differences between the treatments, however, the differences were not significant (Figure 4).

**Figure 5:**
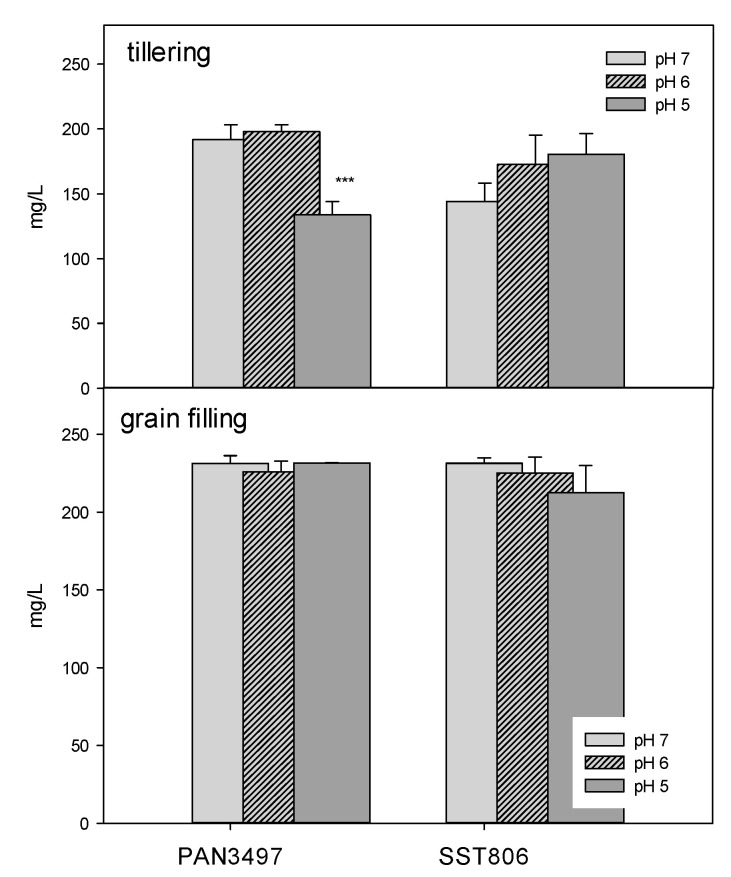
Protein content in leaves of PAN3497 and SST806 grown at three soil pH levels (pH 7, 6, and 5), with two sampling times (tillering and grain filling). Each value is the mean ± standard error of three biological and technical replicates. Significant difference compared to pH 7: ** *p* < 0.001.

Although protein content increased with a decrease in pH for SST806, the treatments were not significantly different at the tillering stage. For PAN3497 at tillering, protein content was significantly decreased at pH 5. In contrast, at grain filling, there were no significant differences between treatments or genotypes (Figure 5).

The cultivar effect was only significant (*p* ≤ 0.01) for MDA content. Based on ANOVA the influence of treatments were significant (*p* ≤ 0.001) on MDA, proline, and protein content and insignificant on APX and POD. Highly significant sampling time effects were measured for all characteristics. POD was the least influenced by the sampling time. The cumulative effects of cultivar and pH treatments significantly influenced the proline and protein content. In addition, a highly significant interaction was observed between cultivar and sampling times for MDA content. Treatment and sampling time interactions were significant for APX, POD, proline, and protein content (Table 1). 

Table 2 shows the average values of the two genotypes. The pH treatments had no effect on POD. APX slightly increased with decreasing soil pH. Decreasing soil pH inversely affected MDA content, which was highest for pH 5. Proline content significantly increased in all treatments, with the largest effect at pH 5. Protein content was the least for pH 5 treatment.

The genotype had no effect on POD. APX activity was insignificantly higher for SST806 compared to PAN3497. PAN3497 had a significantly lower MDA content than SST806, whilst the proline content was notably higher for SST806 compared to PAN3497 (Table 3). Protein content was significantly higher for PAN3497 compared to SST806.

## 3. Discussion

Exposure to abiotic stressors such as acidic soil conditions commonly results in the overproduction of reactive oxygen species (ROS) [31]. The increased activity of antioxidant enzymes is one of the first reactions as a response of plants to oxidative stress [10].

The few studies that have reported on the influence of oxidative stress catalyzed by acidic soil conditions were contradictory. Long et al. [29] stated that low pH influenced ROS more in roots than in leaves. Bhuyan et al. [32] stated that under acidic growing conditions, as well as during other abiotic stresses such as cold stress [33] wheat MDA content was higher. In this experiment, MDA content, APX and POD activity, proline, and protein content of wheat leaves significantly changed when plants were grown in pH 5 soil. Moreover, a very strong cultivar effect was found. Significant changes were observed only at pH 5 in the case of SST806, while MDA concentration and POD activity significantly changed in PAN3497 at soil pH 6. 

Turhan et al. [34] found that the growth stage of a plant influences its response to abiotic stress, which was confirmed by the higher levels of proline, protein, and MDA content at grain filling than at the tillering stage in the current study. Firscher [35] also stated that the most sensitive development stages of wheat are stem elongation, after flowering, and grain filling. Dreccer et al. [36] showed that high temperatures and water imbalance influenced the production of barley, wheat, chickpea, and canola at tillering, stem elongation, and grain filling. 

The effect of cultivar on MDA content was negligible, although SST806 had slightly higher values during grain filling. The sampling time effect was significant for the different traits. Nikolaeva et al. [37] stated that the activity of APX, proline, and MDA content of wheat leaves were influenced by cultivar, time of stress, and the phase of leaf development. This study showed similar results. The effects of the treatments were highly significant for MDA, proline, and protein content in this experiment. MDA and proline content was notably higher in the pH 5 treatment, whilst protein content was reduced compared to pH 7 conditions in this study. MDA content was higher by 95% when wheat was grown at pH 5.5 compared to pH 7.0 conditions [32]. 

Proline is a so-called non-enzymatic antioxidant. Its accumulation is caused by increased synthesis or moderated deterioration [38], which is one of the plants’ reactions to abiotic stressors [39]. In this study, proline content was significantly higher at pH 5.0 compared to pH 7.0 in both cultivars. A higher concentration of proline is an indication that the plants are exposed to stressors [40]. The expression levels of the antioxidant (SOD, CAT, and POX) and proline genes responsible for scavenging or neutralizing ROS were identified in plants. The tolerance of plants to low pH soil is based on the expression of genes related to proline and antioxidant production [41].

Interestingly, proline may enhance the activity of some antioxidant enzymes, for instance, peroxidase [42]. APX activity was also stated to be enhanced under several stress conditions; e.g., Shi et al. [28] found that the activity of APX was higher when cucumber was grown at pH 4.5 compared to pH 6.5. However, the current study did now show significant changes in APX activity. Furthermore, Zhang et al. [22] also published enhanced APX activity in rice roots at pH 2.5. They suggested that the activation of APX is a principal part in scavenging ROS and aids the adaptation of plants to acidic pH. 

According to Sanmartin et al. [43], oxidative stress can catalyze the expression of ascorbate oxidase (AOX), suggesting its role in regulating oxidative stress [44]. In the current study, the APX was significantly higher at 6 pH in PAN3497 and also significantly higher in SST806 at pH 5.0 during the grain filling stage.

In conclusion, growth stages influence the rate of lipid peroxidation, as well as the antioxidative capacity of bread wheat cultivars with a larger effect at grain filling, making this a very important time for studying the effects of acidity stress at the biochemical level. Although SST806 had higher proline, POD, and APX levels, this coincided with a very high MDA content. This shows that the high antioxidative capacity observed here cannot be associated with the reduction of membrane damage under low soil pH. This study should therefore be advanced to establish the role of the induced antioxidant system in association with the yield performance of these cultivars. The current research findings were obtained under controlled conditions in a glasshouse. Nitrogen leaching or other circumstances can affect results in field conditions.

## 4. Materials and Methods

### 4.1. Green House Trial 

Two South-African bread wheat genotypes, PAN3497 and SST806, were sown in 2 L pots filled with 2 kg soil in the greenhouse (temperature was 18 °C during the night and 22–24 °C during the day) in a randomized complete block design with three factors (pH treatments, genotypes, and sampling times) and three repetitions (20 pots per repetition where each pot contained three plants). The soil was collected at Bainsvlei (GPS S 29.05° S 26.11667°, Bloemfontein, South Africa) from 1.5 m deep subsoil. The data of the soil analysis are shown in Table 4. Soil nitrogen content was determined with the classic Kjeldahl-method [45]. Soil phosphorus availability was measured with Bray 1 [46]. The amount of extractable cations and microelements were determined with the use of ammonium acetate, where 2.5 g soil and 25 mL 1 M ammonium acetate was applied for the extraction. The concentration of cations was measured with an atomic adsorption spectrophotometer, based on Miller et al. [47]. The total organic carbon content of the soil was measured by the Walkley–Black method [48]. The soil texture (sand, clay, and silt %) was determined with the Bouyoucos method [49]. To determine the soil density, the weight of 10 cm^3^ of dry soil was divided by its volume. 

The experiment was conducted from June to the end of October 2018.

The following macro and micronutrient fertilization was applied for all treatments: 261 mg L^−1^ KNO_3_, 210 mg L^−1^ K_2_SO_4_, 87 mg L^−1^ NH_4_H_2_PO_4_, 758 mg L^−1^ Ca(NO_3_)_2_, 348 mg L^−1^ MgSO_4_, 3.45 mg L^−1^ C_10_H_13_FeN_2_O_8_, 0.30 mg L^−1^ MnSO_4_, 0.13 mg L^−1^ ZnSO_4_, 0.62 mg L^−1^ H_3_BO_3_, 0.05 mg L^−1^ CuSO_4_, 0.02 mg L^−1^ Na_2_MoO_4_. 

### 4.2. Treatments

For low soil pH treatments, sulfur (S) was applied at different concentrations. The control did not receive any S. The S treatments were as follows: in order to the lower pH to 6, 0.155 g of S was added per kg of soil; to lower the pH to 5, 0.387 g of S was added per kg of soil. 

After the treatments, the soil was kept moist and incubated for four weeks with weekly pH measurements to ensure that the desired pH was reached. Sowing commenced after the soil incubation period.

### 4.3. Sampling 

The youngest leaves were collected from the main stem at the tillering and grain filling stages. Thereafter, they were placed in liquid nitrogen and stored in an ultra-freezer at 70 °C until the determination of the measured parameters. 

### 4.4. Enzymes Assays 

The experiment followed the process from Pukacka and Ratajczak [50] to make the enzyme extracts. Samples (1 g) of each treatment were weighed, homogenized with liquid nitrogen and 5 mL of 50 mM potassium phosphate buffer (pH 7.0) was added. The buffer contained the following chemicals: 1 mM EDTA, 2% PVPP, 0.1% Triton X-100 and 1 mM ascorbate. To obtain the supernatant used for examination, the resulting extract was centrifuged at 15,000× *g* for 20 min at 4 °C. 

The APX assay was carried out based on Mishra et al. [51] with some changes to the composition of the reaction mixture. Each 1 mL of mixture contained 470 μL 50 mM phosphate buffer (pH 7.0), 250 μL 0.1 mM H_2_O_2_, 200 μL 0.5 mM sodium ascorbate, 50 μL 0.1 mM EDTA and 30 μL enzyme. The APX activity was measured from the decrease in absorbance due to ascorbate oxidation. The absorbance was measured at 290 nm for 5 min at 20 °C against a blank, which contained 50 mM phosphate buffer instead of the enzyme. Each sample was measured three times. To calculate the enzyme activity, an extinction coefficient of 2.8 mM^−1^cm^−1^ was applied. 

To measure the POD activity, the study used a method published by Zieslin and Ben-Zaken [52] with some modifications to the amount of chemicals used in the reaction mixture. The modified mixture had 50 μL 0.2 M H_2_O_2_, 100 μL 50 mM guaiacol, 340 μL distilled H_2_O, 500 μL 80 mM phosphate buffer (pH 5.5), and 10 μL enzyme. The POD activity was determined based on the concentration of generated tetraguaiacol. The absorbance of the reaction mixture was read at 470 nm for 3 min at 30 °C. All the above mentioned chemicals were used for the blank, but 50 mM phosphate buffer was used instead of the enzyme. To calculate the concentration of tetraguaiacol created, the extinction coefficient was 26.6 mM^−1^cm^−1^. 

The study used the method of Bradford [53] to measure protein content for the enzyme extract.

The rate of lipid peroxidation was deduced by measuring the quantity of malondialdehyde (MDA) generated in the chemical assay, using the method described by Heath and Packer [54]. Wheat leaves (100 mg) were homogenized with liquid nitrogen, and 1 mL 0.25% thiobarbituric acid (TBA) and 10% trichloroacetic acid (TCA) was added. The samples were centrifuged at 10,800× *g* for 25 min at 4 °C and the supernatants were used. The reaction was generated by the use of 0.2 mL supernatant and 0.8 mL 20% TCA, then 0.5% TBA was added to a clean Eppendorf tube. The mixture was vortexed, heated at 95 °C for 30 min and immediately cooled on ice. This was followed by centrifugation at 10,800× *g* for 10 min at 4 °C. The absorbance was read at 532 nm and 600 nm. The amount of malondialdehyde was computed with the use of an extinction coefficient of 155 mM^−1^cm^−1^.

The proline content in the leaves was measured based on Carillo and Gibon [55]. Fresh wheat leaves (0.1 g) were homogenized with liquid nitrogen and 2 ml 70% (*v*/*v*) ethanol was added [56]. The 1 mL reaction mix (1% ninhydrin in 60% (*v*/*v*) acetic acid) was added to 500 μL ethanolic extract and placed into 1.5 mL Eppendorf tubes, which were shaken and kept in a 95 °C water bath for 20 min. Afterward, samples were cooled on ice and centrifuged at 12,000× *g* for 1 min. The absorbance of the samples was measured at 520 nm, and the amount of proline was calculated from the proline standard curve. The color of our reaction with ninhydrin was yellowish to deep yellow/pinkish, which indicated the presence of proline. We established a proline standard curve using different concentrations of proline in this experiment to verify the content of proline in our extracts. Similarly, the reaction of proline standards were also yellowish and pinkish (depending on the concentration of proline). 

### 4.5. Statistical Analysis

Analysis of variance (ANOVA) was carried out on the data for both genotypes, the four treatments, and two years, as a three-factor analysis [57]. ANOVA was also used for the two cultivars separately, as well as for the two sampling times combined, in order to observe the influence of treatments on the APX, POD, MDA, proline, and protein contents within each cultivar. Differences were inspected at a *p* < 0.05 level of significance. The Tukey test and least significant difference (LSD) were used for means separation. 

## Figures and Tables

**Table 1:** The mean squares from the combined analysis of variance for ascorbate peroxidase activity, malondialdehyde content, peroxidase activity, proline, and protein content for two wheat cultivars, with three treatments over two sampling times.

	Cultivar (C)	Treatment (T)	Sampling Times (S)	CxT	CxS	TxS	CxTxS
APX	1.91	1.58	281.91 ***	0.75	0.03	4.17 **	0.68
MDA	441.39 **	1055.19 ***	1144.73 ***	20.67	571.61 **	181.34	28.36
POD	0.00	0.01	0.30 ***	0.01	0.01	0.06 **	0.01
Proline	612.26	17,677.81 ***	185,844.69 ***	1038.73 *	2003.23	15,533.44 *	833.11
Protein	485.68	1905.05 *	62,151.63 ***	2011.45 **	679.57	4362.51 ***	3423.66 ***

* *p* < 0.05; ** *p* < 0.01; *** *p* < 0.001; APX, ascorbate peroxidase; MDA, malondialdehyde; POD, peroxidase.

**Table 2:** Average values of two genotypes and two sampling times for measured parameters for three treatments.

	pH 7 Mean ± S.D.	pH 6 Mean ± S.D.	pH 5 Mean ± S.D.	LSD (0.05)
APX	3.32 ± 2.03	3.71 ± 2.30	3.78 ± 2.56	0.38
MDA	26.51 ± 4.32	28.05 ± 6.60	38.76 ± 10.42	3.71
POD	0.40 ± 0.03	0.41 ± 0.05	0.41 ± 0.14	0.03
Proline	57.10 ± 38.22	64.46 ± 53.17	102.17 ± 83.80	8.54
Protein	199.60 ± 41.41	206.00 ± 23.03	189.10 ± 42.93	9.65

Values in columns are means ± standard deviation; APX, ascorbate peroxidase; MDA, malondialdehyde; POD, peroxidase.

**Table 3:** Average values for measured parameters in two genotypes with three treatments over two sampling times.

	PAN3497	SST806	LSD (0.05)
APX	3.36 ± 2.04	3.84 ± 0.11	0.31
MDA	29.03 ± 6.26	33.19 ± 11.14	3.03
POD	0.40 ± 0.05	0.41 ± 0.11	0.03
Proline	72.58 ± 58.97	76.57 ± 65.08	6.97
Protein	202.00 ± 37.36	194.97 ± 33.64	7.88

Values in columns are means ± standard deviation; APX, ascorbate peroxidase; MDA, malondialdehyde; POD, peroxidase.

**Table 4:** Main parameters of the used soil.

Parameters	Value
pH_H2O_	6.8
sand (%)	96
clay (%)	4
silt (%)	0
CEC (cmol _C_ kg^−1^)	3.27
density (g cm^−1^)	1.18
C (%)	0.04
N (mg kg^−1^)	<0.004
P (mg kg^−1^)	26.5
K (mg kg^−1^)	163.6
Ca (mg kg^−1^)	13,630 *
S (mg kg^−1^)	1.62
Mg (mg kg^−1^)	38.914 *
Na (mg kg^−1^)	1.80 *
Fe (mg kg^−1^)	4.82
Zn (mg kg^−1^)	0.71
Cu (mg kg^−1^)	0.60
Mn (mg kg^−1^)	21.39

* result as a % of CEC. CEC, cation exchange capacity.
